# Current methodological and technical limitations of time and volumetric capnography in newborns

**DOI:** 10.1186/s12938-016-0228-4

**Published:** 2016-08-30

**Authors:** Gerd Schmalisch

**Affiliations:** Department of Neonatology, Charité University Medical Center, Charitéplatz 1, 10117 Berlin, Germany

**Keywords:** Capnography, End-tidal CO_2_, Dead space, Tidal volume, Respiratory function monitoring, Newborns

## Abstract

Although capnography is a standard tool in mechanically ventilated adult and pediatric patients, it has physiological and technical limitations in neonates. Gas exchange differs between small and adult lungs due to the greater impact of small airways on gas exchange, the higher impact of the apparatus dead space on measurements due to lower tidal volume and the occurrence of air leaks in intubated patients. The high respiratory rate and low tidal volume in newborns, especially those with stiff lungs, require main-stream sensors with fast response times and minimal dead-space or low suction flow when using side-stream measurements. If these technical requirements are not fulfilled, the measured end-tidal CO_2_ (*P*_*et*_*CO*_*2*_), which should reflect the alveolar CO_2_ and the calculated airway dead spaces, can be misleading. The aim of this survey is to highlight the current limitations of capnography in very young patients to avoid pitfalls associated with the interpretation of capnographic parameters, and to describe further developments.

## Background

**Fig. 1:**
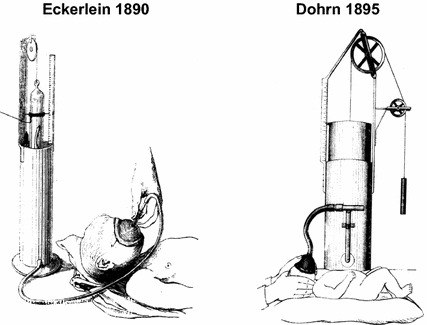
First ventilatory measurements in newborns using custom made mechanical spirometers [4, 5]

Acute respiratory disorders in newborns are an important clinical problem, especially in preterm infants [1]. Premature birth interrupts normal in utero lung development, resulting in significant alterations in postnatal lung function with consequences in later life [2, 3]. Clinical interest in postnatal lung function measurements at the end of the 19th century resulted in the development of special mechanical spirometers (Fig. 1) to measure tidal volume (*V*_*T*_), respiratory rate (*RR*) and minute ventilation (*V’*_*E*_) [4, 5]. These mechanical measuring systems, however, have limitations in newborns, the most important being the high ratio of apparatus dead space (*V*_*Dapp*_) to tidal volume (*V*_*T*_), resulting in CO_2_-rebreathing and a risk of hypercapnia.

Comprehensive investigations of postnatal lung function, providing insights into neonatal respiratory physiology and pathophysiology, were first performed in the 1950s, after the development of electronic devices suitable for measuring and recording small and fast respiratory signals [6–8]. Besides measuring ventilation, respiratory mechanics and lung volume, the non-invasive measurements of the exhaled carbon dioxide (CO_2_) was at the beginning in the focus of the pulmonary researcher because its crucial role for the assessment of the alveolar gas exchanges and airway dead spaces (*V*_*D*_). The measured CO_2_-signal can be recorded as a function of time (time-based capnography, mostly used for monitoring purposes) or volume (volumetric capnography), which allows calculations of alveolar ventilation (V’_A_) and airway dead spaces.

Since the 1960s different types of commercial capnographs have been developed for continuous CO_2_ measurements in adults, and these devices have been adapted for measurements in neonates [9, 10]. The first capnographs were bulky and cumbersome to use, especially when coupled to mass spectrometry [11]. During this time, a new generation of capnographs was developed for measurements in infants, with these instruments resolving many problems associated with earlier capnographs. Lightweight infrared (IR) mainstream sensors with a dead space <1 mL enabled reliable measurements even in preterm infants [12–14]. In addition, special low-flow sidestream capnographs were developed for dead space free measurements in neonates, making long-term monitoring possible [15–17]. Several clinical studies have shown the clinical benefit of the time-based capnography in ventilated infants and children, e.g., for non-invasive monitoring of the arterial pCO_2_ [18–20], to verify endotracheal tube placement [21–23], and to monitor the integrity of the ventilator circuit including disconnection and accidental extubation [24–26]. In spontaneously breathing infants time-based and volumetric capnography has been used for assessment of functional lung alterations related to bronchopulmonary dysplasia (BPD) [27–29]. Nevertheless the clinical use of capnography in newborns with the equipment available today still has methodological and technical problems which limits its wide adoption as a routine tool in the neonatal intensive care. This survey highlights the current limitations of capnography in these very young patients to avoid pitfalls associated with the interpretation of capnographic parameters which can lead to false clinical decisions, and to describe further developments.

## Peculiarities of capnography in small lungs

The lungs and breathing patterns of neonates differ in many respects from those in adults. These differences may alter CO_2_ measurements as well as the interpretation of time-based and volumetric capnograms and derived parameters [30, 31]. Furthermore, the impact of the patient interface on CO_2_ measurements in breathed air and on the homogeneity of the alveolar ventilation [32] is much higher in neonates than in adults, as neonates have a much higher ratio of *V*_*Dapp*_ to tidal volume [12, 33]. Thus, difficulties in capnographic measurements are greater in smaller infants.

### Ventilatory parameters

The measured ranges of the CO_2_ fraction (*FCO*_*2*_) and the corresponding partial pressure (*PCO*_*2*_) in exhaled air are identical in neonates and adults. However, CO_2_ production is much lower in neonates (about 15 mL/min) than in adults (about 200 mL/min), and is even lower (9 mL/min) in very low birth weight (VLBW) infants, defined as those with a birth weight <1500 g [34]. The much lower amount of exhaled CO_2_ makes capnography in neonates more difficult, because there are objective limits in reducing the size of the analyzer chamber to realize fast filling when using a mainstream sensor or in reducing the magnitude of suction flow by side-stream measurements.

The ratio of tidal volume to body weight of a term infant is about 7 mL/kg, nearly the same as in adults. This ratio is slightly lower in preterm infants, 5–6 mL/kg. VLBW infants are at higher risk of having respiratory diseases and needing mechanical respiratory support and may have tidal volumes <10 mL. This low tidal volume limits the accuracy of capnographic measurements in these infants, given that the *V*_*Dapp*_ of current mainstream gas analyzers is about 1 mL. Another limitation is the high respiratory rate in these infants. Adults have a breathing rate of 10–12/min, term newborns of 40–45/min and preterm newborns of 55–60/min. Furthermore, preterm infants with respiratory deficiency can have a respiratory rates >60/min with exhalation times of <500 ms [35, 36]. Energetically, this is an optimal breathing strategy for infants with stiff lungs, as it reduces the work of breathing [37]. However, this breathing strategy results in a technical challenge in capnographic measurements using currently available equipment.

### Small airways

**Fig. 2:**
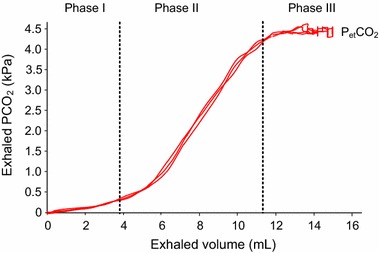
Typical volumetric capnogram of a ventilated preterm infant with a wide phase II and a short phase III, which defined the end-tidal CO_2_ (*P*_*et*_ *CO*_*2*_)

**Fig. 3:**
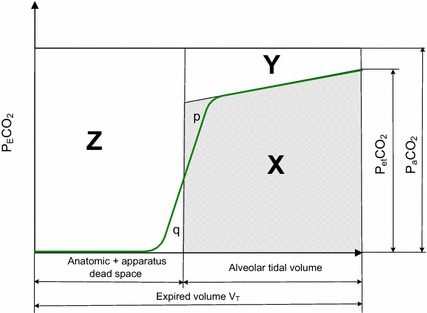
Dead space calculation by Fletcher’s method using Fowler’s equal areas (p = q) to define dead space. The area *X* corresponds to the exhaled amount of CO_2_ and the areas *Z* and *Y* to the nonexhaled CO_2_ as a consequence of the anatomic + apparatus (*X*) and alveolar (*Y*) dead spaces

Both the lungs and airways grow in volume from infancy to adulthood [38], with the upper airways contributing significantly to anatomic dead space visible in volumetric capnograms. Measurements on volumetric capnography can be divided into three phases, as shown in Fig. 2. Phase I represents the CO_2_ free interval due to anatomic and apparatus dead space; phase II is characterized by a rapid increase in CO_2_ and represents the transition between airway gas and alveolar gas; and phase III reflects alveolar gas at the alveolar plateau, which is terminated by the most-well known capnographic parameter, the end-tidal CO_2_ (*P*_*et*_*CO*_*2*_) which is commonly lower than the arterial PCO_2_ (*P*_*a*_*CO*_*2*_) due to the alveolar dead space ventilation as shown in Fig. 3. In healthy adult lungs, CO_2_ concentration rapidly increases during phase II because the upper airways contribute negligibly to gas exchange and sophisticated techniques were developed to evaluate and classify time-based and volumetric capnograms [39, 40]. In neonates, airway diameters are much smaller and the ratios of inner surface area and volume per unit length are much higher than in adults, resulting in gas exchange between gas and tissue in the airways. This may result in an exaggerated and not well defined phase II and a reduced or even missing phase III in neonates which hampers the quantitative evaluation. Using volumetric capnography Ream et al. [41] found an inverse relationship between the slope of the alveolar plateau and infant size. Moreover, in a study by Fouzas et al. [5] phase III was found to be likely steeper in infants with than without BPD also due to airway obstruction that occurs in this disease process, suggesting that BPD likely induces a ventilation/perfusion mismatch in the lungs. Thus, in premature infants and infants with respiratory diseases, it is much more difficult to differentiate between phase II and III in the volumetric capnogram and to define an alveolar plateau. This result is supported by modeling studies by Schwardt et al. [42] and Neufeld et al. [43], which demonstrated an association between a morphometric reduction in airway cross-section and increased diffusional resistance within the airways, resulting in an increase in the slope of phase III. As phase III becomes steeper, it becomes more difficult to define an alveolar plateau. The nomenclature of the volumetric capnogram is also used for time-based capnograms, however, the interpretation is not equivalent. An alveolar plateau in a time based capnogram does not give any information if and how much alveolar CO_2_ was exhaled [31]. Nevertheless a missing or falsified alveolar plateau in the time-based and volumetric capnogram may indicate that the measured *P*_*et*_*CO*_*2*_ does not reflect the alveolar CO_2_ pressure, making it problematic to use the measured *P*_*et*_*CO*_*2*_ to monitor blood gases [44, 45]. Therefore, *P*_*et*_*CO*_*2*_ measurements are only useful if the shape of the capnogram is considered. Automated measurements of *P*_*et*_*CO*_*2*_ without monitoring the waveform should be used with caution, as the resulting data can be misleading.

### Missing alveolar plateau

In newborns, volumetric capnograms without alveolar plateaus are not uncommon. A clinical study in spontaneously breathing preterm infants by Tirosh et al. [30] showed that the number of capnograms without alveolar plateaus increased significantly with decreasing gestational age. Proquittè et al. [31] investigated the effects of lung stiffness on the incidence of capnograms without alveolar plateaus in 21 ventilated newborn piglets (body weight 560–1435 g) before and after lung lavage with saline. They found that, in healthy lungs, 10 % of capnograms did not show an alveolar plateau, whereas the incidence in surfactant-depleted lungs was about 50 %. Furthermore, the incidence of capnograms without an alveolar plateau increased markedly as exhalation time decreased, with >75 % of capnograms lacking an alveolar plateau when exhalation times were <200 ms. Although the cause remains unclear, they likely result from both the technical limitations of the current technique (primarily, the overly long response time) and the physiological peculiarities of CO_2_ exchange in small stiff lungs.

### Airway dead spaces

In adult lungs, only alveolar ventilation takes part in gas exchange with pulmonary capillary blood, while gas exchange in the physiologic dead space (*V*_*Dphys*_) is negligible. *V*_*Dphys*_ comprised the alveolar dead space (*V*_*Dalv*_) of non-perfused or under-perfused alveoli, the anatomic dead space (*V*_*Dana*_) of the conducting airways and the apparatus dead space (*V*_*Dapp*_) of the face mask, pneumotach and mainstream gas analyzer. As early as the 1960s, Chu et al. [46] used in neonates a rapid capnograph to measure P_et_CO_2_ in exhaled air, with the anatomic and physiologic dead space calculated using the Bohr equation:

1 $$ V_{Dana} \; = \;V_{T} \frac{{P_{et} CO_{2} - P_{mean} CO_{2} }}{{P_{et} CO_{2} }} $$

or the Bohr/Enghoff equation by substituting *P*_*a*_*CO*_*2*_ for *P*_*et*_*CO*_*2*_:

2 $$ V_{Dphys} \; = \;V_{T} \frac{{P_{a} CO_{2} - P_{mean} CO_{2} }}{{P_{a} CO_{2} }} $$

where mean *P*_*mean*_*CO*_*2*_ is the mean CO_2_ tension of the mixed expired air. The difference between these dead spaces represents the alveolar dead space:

3 $$ V_{Dalv} \; = \;V_{Dphys} \; - \;V_{Dana} $$

The measured *V*_*Dana*_ includes the dead space of the equipment. The Bohr and Bohr/Enghoff equations use only three measured parameters for dead space calculations, independent of the shape of the capnogram. Fletcher et al. [47] were the first who developed the calculation of different dead spaces from volumetric capnograms shown in Fig. 3. The three dead spaces can be calculated by ratios of the areas *X, Y,* and *Z* using Fowler’s classic ‘equal area’ method (*p* = *q*) [48].

$$ V_{Dphys} = V_{T} \frac{Y + Z}{\begin{aligned} X + Y + Z \hfill \\ \hfill \\ \end{aligned} } $$

$$ V_{Dalv} = V_{T} \frac{Y}{\begin{aligned} X + Y + Z \hfill \\ \hfill \\ \end{aligned} } $$

4 $$ V_{Dana + app} = V_{T} \frac{Z}{\begin{aligned} X + Y + Z \hfill \\ \hfill \\ \end{aligned} } $$

Fletcher’s method requires a clear subdivision of the capnogram into phases I, II and III. It is, therefore, often difficult to apply this method to measurements in small lungs, in which phases II and III are not well defined. This method was inapplicable in the absence of an alveolar plateau. A clinical study of ventilated infants with a median birth weight of 2658 g showed that Fletcher’s method failed in 67 %, especially in preterm infants, whereas the Bohr/Enghoff equations could be used in all [12]. Therefore, in small infants the Bohr/Enghoff equation has been preferentially used to calculate dead space [44, 49, 50]. However, this advantage may be deceptive, because the extent to which phase III steepness or a missing alveolar plateau leads to errors in calculating dead space has not been determined. The more a capnogram differs from a step function, the more unreliable will be the dead-spaces calculated by Bohr/Enghoff equations.

### Endotracheal tube leaks

The time-based capnography is widely used in both the intensive care unit (ICU) and during anesthesia to monitor gas exchange and the integrity of the ventilator circuit [51, 52]. In contrast to adults, newborns are frequently intubated with uncuffed endotracheal tubes (ET) to protect the airways and to avoid subglottic stenosis [53, 54]. Uncuffed ETs, however, have been associated with a risk of inadvertent air leaks [55], which occur in about 70 % of ventilated neonates [56]. In contrast to tidal volume measurements [57, 58], the effects of ET leaks on measurements of exhaled CO_2_ are much more complex [59]. At the end of expiration, when patient flow is zero, an ET leak can lead to reverse flow through the sample chamber, washing out the exhaled CO_2_ and resulting in a measured *P*_*et*_*CO*_*2*_ close to zero. Leak-dependent CO_2_ measurement errors depend on the shape of the CO_2_ plateau in exhaled air [59]. As long as the CO_2_-plateau of exhaled air reaches the sample chamber before reverse flow starts, the peak CO_2_ will represent the *P*_*et*_*CO*_*2*_. Therefore, in contrast to volume measurements [58, 60], it is not possible to determine an upper limit of ET leaks that may be tolerated clinically for capnographic measurements. In mechanically ventilated preterm infants with low compliant, stiff lungs the frequently very short or absent CO_2_ plateaus may be notably falsified by the leak. If ET leakage occurs, capnographic measurements in these infants should be interpreted with caution, because of the uncertainty of *P*_*et*_*CO*_*2*_.

## Technical limitations

### Apparatus dead space

The apparatus dead space is of particular interest for capnographic measurements because it can lead to rebreathing of exhaled CO_2_, thereby generating false inspiratory and expiratory CO_2_ measurements. This was a significant problem using volumetric capnography with serial connections of the CO_2_ analyzer and pneumotach [61]. Combined sensors with a dead-space of about 1 ml have become available, e.g., neonatal sensor of the NM3, NICO, and CO_2_SMO+ respiratory monitors (all from Philips-Respironics, Murrysville, PA, USA). Nevertheless, the dead space problem remains, especially in preterm infants with low tidal volumes.

Dead space-free mainstream capnography remains a technical challenge, similar to that of dead space-free ventilatory measurements by the flow-through technique, in which the apparatus dead space is virtually eliminated by a background flow [62]. Evans et al. [63] performed capnographic measurements in 20 one-day old newborns using a bias flow of 3 L/min to wash-out the apparatus dead space of the face mask [63]. However, the bias flow diluted the CO_2_ concentration of the analyzed breathed gas, causing a loss in the precision of CO_2_ measurements, depending on the resolution of the capnograph.

### Response time

Because the respiratory rate is about 4–5 times higher in neonates than in adults, a capnograph should have a shorter response time in neonates than in adults. The response time of the capnograph, however, must be sufficiently short, so that the magnitude and shape of the CO_2_ signal pattern are as accurate as possible. The response time of a capnograph has two components, the transit time and the rise time [64]. In sidestream measurements, the transit time is the time taken by the gas sample to travel from the sample port to the CO_2_ analyzer. This time depends on the suction flow and the length and diameter of the tube, with a numerical correction of the time lag being only approximate. The rise time is the time taken by the CO_2_ analyzer to increase from 10 to 90 % of the final value. Capnographs used in neonates currently have rise times (*T*_*10*–*90* *%*_) of 50–80 ms, depending on the airflow used for testing. This time may be too long for preterm neonates with low expiratory air flow and expiratory times <500 ms. Unfortunately, signal filtering has marginal effects on improving rise times, with improvements limited by the rapid reduction in signal-to-noise ratio [65].

**Fig. 4:**
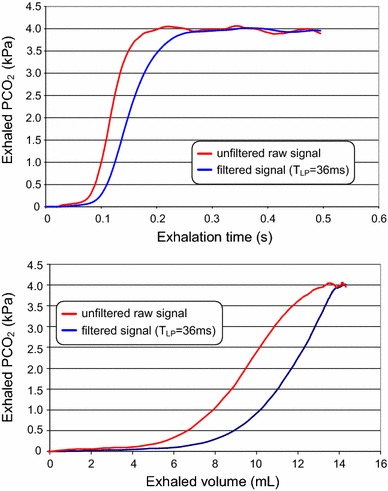
Computer simulation data illustrating the effects of delayed CO_2_ measurements on time based (*top*) and volume based (*bottom*) capnography using a low-pass filtering of the CO_2_ signal (time constant *T*_*LP*_ = 36 ms)

The effects of increased rise time on time-based and volume-based capnograms are shown in Fig. 4. A CO_2_-signal of a ventilated newborn with a short exhalation time was low pass filtered to simulate an increase in the rise time of the analyzer. The time constant of 36 ms is equivalent to a T_10–90 %_ of 80 ms. Although the errors in *P*_*et*_*CO*_*2*_ are relatively small, low pass filtering shifts the volumetric capnogram to the right, resulting in overestimates of calculated dead spaces which can lead to misleading clinical interpretations [66]. Thus, dead space measurements in neonates with short exhalation times are much more sensitive to the rise time of the CO_2_-analyzer than *P*_*et*_*CO*_*2*_ measurements. Therefore, the results of older studies in newborns, using capnographs developed for adults with much higher rise times, should be interpreted with caution.

**Fig. 5:**
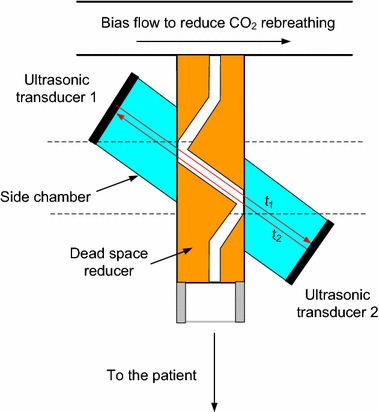
Schematic diagram of an ultrasonic flow meter (Exhalyzer D, Ecomedics, Dürnten, Switzerland). The flow rate *V′(t)* is directly proportional to the difference between transit times *t1* and *t2*. The molar mass of the gas can be calculated from the sum of *t1* and *t2*

Ultrasonic flowmeters that simultaneously measure air flow and the molar mass of breathed gas [67] have very short response times, with no time delays between the two signals (Fig. 5). Although theoretically well suited to dead space measurements, molar mass is a surrogate signal, making it difficult to separate the CO_2_ signal [68]. Neumann et al. [69] used an ultrasonic flowmeter with an additional *V*_*Dapp*_ of 1.1 mL (Exhalyzer D, Ecomedics, Dürnten, Switzerland) for capnographic measurements in mechanically ventilated preterm infants. However the weight and size of currently available ultrasound flow sensors are still too large for widespread clinical use in ventilated neonates.

### Sampling rate

In the past capnographs developed for adults have often been shown inappropriate for measurements in neonates. Besides the high suction flow when using sidestream measurements, the long rise time and the large volume of the analyzer chamber, the sampling rates of analogue-to-digital (A/D) converters were often too low for fast respiratory signals [66, 70, 71]. Furthermore, it is crucial that the analogue flow and CO_2_ signals pass through anti-aliasing filters prior to sampling, in order to satisfy the Shannon sampling theorem and to avoid the potentially insidious problems of aliasing. This requires the specification of appropriate frequency cut-offs, which may differ between adults and newborns [72].

Beside the frequency cut-offs of the anti-aliasing filter the necessary sampling rate depends also on the frequency content of the signals. For acquisition of tidal breathing data, particularly in rapidly breathing infants with low respiratory time constants sampling rates of ≥200 Hz are recommended [72]. The frequency content of the CO_2_ signal depends on the rise time of CO_2_ sensor and is still unknown for infants with low respiratory time constant. The rise time of the current infrared CO_2_ sensors is likely too high to measure correctly the dynamic of CO_2_ signals in stiff lungs with exhalation times <200 ms (Fig. 4). A sampling rate of 100 Hz has been shown to be normally adequate in mature infants when calculating only *V*_*T*_*, P*_*et*_*CO*_*2*_ and airway dead spaces by the Bohr/Enghoff equations. Greater time resolution may be required in rapidly breathing infants or intubated infants with stiff lungs, provided that faster CO_2_ analyzer are available. Furthermore, in contrast to time-based capnograms, the distances between sampling points in volumetric capnograms are not equidistant, reducing the ability to cleanly distinguish among phases I, II, and III of volumetric capnograms. A sufficiently high digital resolution is also necessary for graphical displays of time- and volumetric capnograms, because visual assessment of a capnogram is essential for valid interpretation of capnogram-derived parameters and for calculating interactive dead space by Fletchers method [61].

### Further technical developments

The smaller tidal volumes and higher respiratory rates encountered in neonates, compared with those in older infants and adults, result in higher demands on capnograph use in neonates and require further developments [73]:

- Minimizing the dead-space of main-stream sensors because of low tidal volume,
- Reducing suction flow of side-stream monitors because of low breathing flow,
- Reducing the response time of the CO_2_ analyzer because of the short exhalation times, especially in preterm neonates with stiff lungs,
- Increasing the sample rate to provide sufficient numerical and graphic resolution of the capnogram, especially in infants with high respiratory rates, and
- Minimizing the phase shift between CO_2_ and flow signals to prevent errors in dead space calculations.

Capnography for ventilated neonates will not be widely accepted by neonatologists as long as capnography is not integrated into the neonatal ventilator without increasing of *V*_*Dapp*_ by the CO_2_-analyzer. This requires from the ventilator manufacturers to develop new combined sensors for air flow and CO_2_ measurements.

## Conclusion

Capnography is a fascinating, noninvasive method for collecting information about breathing, alveolar gas exchange and airway dead spaces. In mechanically ventilated infants it is useful for monitoring the integrity of the ventilator circuit for early detection of mishaps, such as accidental tracheal extubation and disconnection of the breathing circuit, before irreversible damage is caused by prolonged hypoxia. Capnography, however, has physiological and technical limitations in neonatal patients, especially in newborns with stiff lungs. The short exhalation times, low tidal volumes and high impact of apparatus dead space hamper its measurements. Dead space calculations developed for adult lungs are often inapplicable to neonates with a prolonged phase II and a reduced or absent phase III. Imaginative physiological concepts are needed to interpret capnograms in these patients.

Despite technological progress, there are still technical limitations in correctly measuring the fast CO_2_ signals of neonates. These patients require need faster CO_2_ sensors and low suction flow for side stream measurements. Moreover, when using volumetric capnography, the pneumotach should be integrated with the CO_2_ sensors to reduce apparatus dead space. Finally, the widespread acceptance of capnography for neonates requires new, well designed bench, animal, and clinical studies to demonstrate its clinical value and various diagnostic possibilities in these patients.

## Abbreviations

A/D: analogue-to-digital
BPD: bronchopulmonary dysplasia
ET: endotracheal tube
*FCO*_*2*_: CO_2_ fraction
*P*_*et*_*CO*_*2*_: end-tidal CO_2_ partial pressure
*RR*: respiratory rate
*T*_*10*–*90* *%*_: sensor rise time
*V’(t)*: air flow signal
*V(t)*: volume signal
*V’*_*A*_: alveolar ventilation
V’_E_: minute ventilation
*V*_*D*_: dead space
*V*_*Dalv*_: alveolar dead space
*V*_*Dana*_: anatomic dead space
*V*_*Dapp*_: apparatus dead space
*V*_*Dphy*s_: physiologic dead space
*V*_*T*_: tidal volume
VLBW: very low birth weight
